# A nocturnal rail with a simple territorial call eavesdrops on interactions between rivals

**DOI:** 10.1371/journal.pone.0197368

**Published:** 2018-05-18

**Authors:** Lucyna Ewa Wojas, Paweł Wojciech Podkowa, Tomasz Stanisław Osiejuk

**Affiliations:** 1 Department of Behavioural Ecology, Adam Mickiewicz University, Poznań, Poland; 2 Department of Avian Biology and Ecology, Adam Mickiewicz University, Poznań, Poland; Cornell University, UNITED STATES

## Abstract

The behaviour of most animals has evolved in a communication network environment, in which signals produced by senders are perceived by many intended and unintended receivers. In this study, we tested whether the corncrake (*Crex crex*), a nocturnal rail species with innate (non-learned) calls, is able to eavesdrop on the interactions of conspecific males and how this eavesdropping affects subsequent responses by the eavesdropper to territorial intrusion. In the first step, simulated aggressive or neutral interactions between male dyads were presented to a focal male. In the second step, the calls of winning, losing or neutral males from the first step were played within the territory of the focal male. We measured behavioural and vocal responses of focal males. We found that corncrakes eavesdropped on signal exchange between rivals. Males often began responding to distant aggressive interactions during the eavesdropping phase, and they responded strongly during the intrusion phase of the experiments. The response was significantly weaker to playback of males from neutral interactions than to those involved in aggressive interactions, and we found no differences between the responses to Winners and Losers entering a focal male territory.

## Introduction

Communication involves a sender using signals to influence the behaviour of a receiver [1], and living in a social environment requires observation of the members of the society. Therefore, it has been postulated that communication evolves in the context of many simultaneously active senders and receivers coexisting in communication networks [2, 3, 4]. One of the key features of communication networks is eavesdropping [5], which is especially useful for territorial species because it provides information about opponents before an interaction occurs [5]. Social eavesdropping is a behaviour by which an individual obtains information from interactions between other individuals without participating [6] and responds accordingly, thus lowering the cost of territorial defence and minimizing the risks associated with fighting [7, 8]. There are a number of examples of social eavesdropping in many animal taxa, such as fish [9, 10, 11], birds [12, 13], cetaceans [14, 15], humans [16] and even crayfish [17, 18]. In birds, descriptions of social eavesdropping are biased towards songbirds, i.e., Oscines, which are species that acquire song through learning. For example, female great tits (*Parus major*) eavesdrop on vocal interactions between males to choose the best partners for extra-pair copulation [19, 20], while male great tits use information about the hierarchical rank of a potential rival in future interactions [21, 22]. Female black-capped chickadees (*Poecille atricapillus*) whose partners lost in a vocal interaction with an opponent had fewer offspring than females with winning partners [23, 24, 25], and in common nightingales (*Luscinia megarhynchos*), males are able to recognize the motivation of two interacting rivals and respond more strongly to aggression [26]. These studies distinctly demonstrate how social eavesdropping plays a key role for both sexes in songbirds, but to the best of our knowledge, there is only one example of social eavesdropping in non-learning birds, the little blue penguin (*Eudyptula minor*) [27]. Males of this species are able to eavesdrop and modify their behaviour towards the Winners and Losers of simulated vocal exchanges as well as respond to the presentation of displays of triumph after physical fights.

The evolution of acoustic communication in birds was a complex process resulting in both differences in structures responsible for vocal production (brain and vocal production apparatus) among taxa and diversity of vocalization complexity [1, 4, 28]. These differences are clearly visible between learning and non-learning bird species, and song learning is crucially dependent on social interactions and may strongly affect later interactions among neighbours including potential benefits from eavesdropping [29]. Therefore, we assume that eavesdropping may differ between learning and non-learning species, or at least its evolution might be shaped by different constraints in these groups. However, our knowledge of communication networks and social eavesdropping remains insufficient and is strongly biased toward a few learning species that have been the focus of detailed studies.

**Fig 1:**
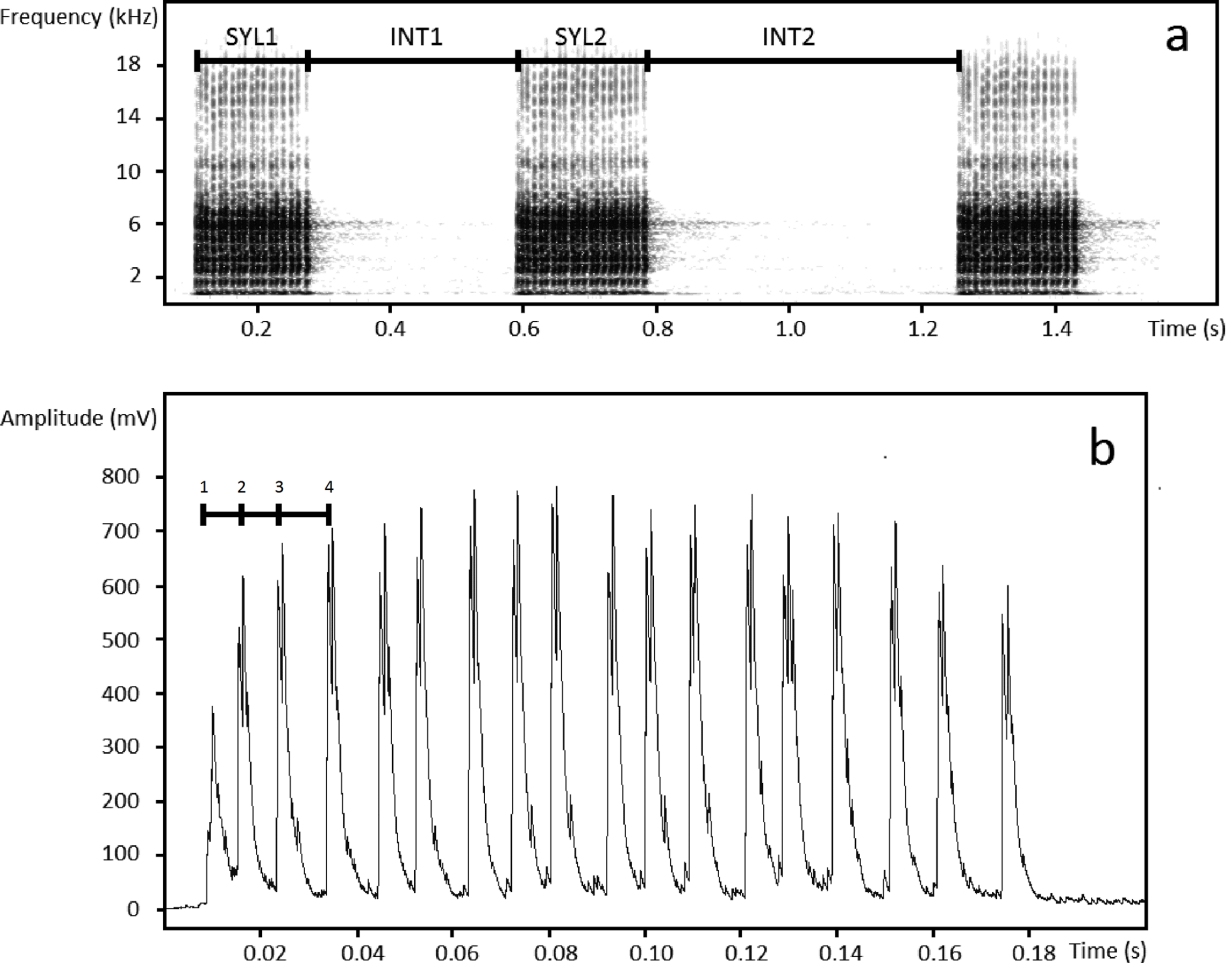
Spectrogram of a corncrake broadcast call. (A) The corncrake call consists of two syllables (SYL1 and SYL2) separated by an interval (INT1), and the calling rhythm is defined as RHYTHM = INT2 / (SYL1 + INT1 + SYL2). (B) An envelope of a broadcast call syllable showing pulse-to-pulse duration, which is used for individual discrimination, with the first 4 pulses marked.

The corncrake (*Crex crex*) is an ideal non-learning bird species for the study of social eavesdropping. This nocturnal, cryptic species communicates almost exclusively through the acoustic channel (acoustic communication in especially useful due to lack of other channels of communication)[30], and males are territorial during the breeding period, during which they advertise their positions with loud calls (referred to as broadcast calls) and behave aggressively toward conspecific rivals [30].The territorial call of the corncrake is very loud (±96 dB SPL at 1 m) and can be heard up to 1 km away [31]. It consists of two syllables repeated at shorter or longer intervals that give the impression of a characteristic rhythm (defined by Osiejuk [32]) that changes from monotonous to intermittent (Fig 1). The rhythm of the call was experimentally demonstrated to function as a conventional signal of aggression, and it provides long-range information about the motivation of the signaller to defend its territory [33]. Males signalling a high motivation similar to that of conspecifics were found most likely to escalate conflicts, which often ended with physical confrontation [33, 34]. Before an attack, males also produce low-amplitude calls with a completely different structure (referred to as soft calls) and a strictly aggressive character [35]. Soft calls are quieter than broadcast calls (70 ± 5 dB SPL at 1 m) and can be heard from 40 m away [35]. The senders of soft calls are more likely to attack, whereas the receivers of soft calls are more likely to either attack or retreat depending on their motivation [34]. Corncrake males were also demonstrated to distinguish between neighbours and unknown intruders by calls and to respond more aggressively to strangers [36]. Hence, the corncrake is a well-recognized model of territorial conflicts involving different types of functional calls and relatively frequent physical attacks (including attacks on playback speakers) that enable the level of aggression to be straightforwardly determined [33, 34]. Moreover, the syllables of calls have an individually specific structure, making it possible to recognize individual calling males based on recordings, even at a distance of up to 300 m (see Fig 1 [36, 37, 38]). The above mentioned characteristics make the corncrake a model bird species to study communication networks, for whom eavesdropping should be extremely beneficial in territorial context. The males are aggressive and readily fight, therefore the information about motivation and fitness of a possible opponent is crucial. Such information is most easily and safely acquired through social eavesdropping.

In this study, we used four loudspeakers to imitate natural interactions between two males in a two-part experimental design. In the first part, we simulated a male-male interaction ending with a clear Winner and Loser or a neutral outcome with no obvious victor at a distance from a focal individual. The Winner always stayed after the end of simulated interaction while the Loser become silent and flew away. In the second part, we simulated an intrusion into the territory of a focal male by playing back the call of the Winner or Loser or a randomly chosen male from the neutral interaction. We predicted that males using social information adjust their behaviour depending on the dominance status of an eavesdropped male to reduce the cost of protecting their territory; in particular, they should adjust the strength of their response in relation to the individual (Winner or Loser of the preceding interaction) entering the territory. Finally, we compared the strength of the responses of males after eavesdropping to the regular responses of males to strangers and neighbours that were tested in earlier experiments [36]. The aim of our study was to experimentally determine if the communication among corncrake males has the characteristics of a communication network.

## Materials and methods

### (a) Study area and subject birds

The study was carried out in the Upper Nurzec River Valley in Northeast Poland (centre of the study area: 52°61’N, 23°20’E) from the 19^th^ May through 16^th^ June, 2013–2015. The study area (ca. 55 km^2^) consisted of meadows, marshes and arable fields, throughout which corncrakes were commonly but irregularly distributed. The basic parameters of abundance and habitat preference are well known from earlier studies [39]. The study subjects were 75 territorial males that were individually recognizable by measurements of the intervals between the maximal amplitude peaks in their calls, i.e., the pulse-to-pulse duration (hereafter PPD; Fig 1). The PPDs of males are individually distinct and stable over the long term, which enables individuals to be recognised without being caught [37, 38]. The study was conducted in full compliance with the current laws of the Poland. All research activities described in this paper were approved by Regional Directorate for Environmental Protection in Białystok, permit no. WPN.6401.251.2012.WL.

### (b) Experimental protocol

**Fig 2:**
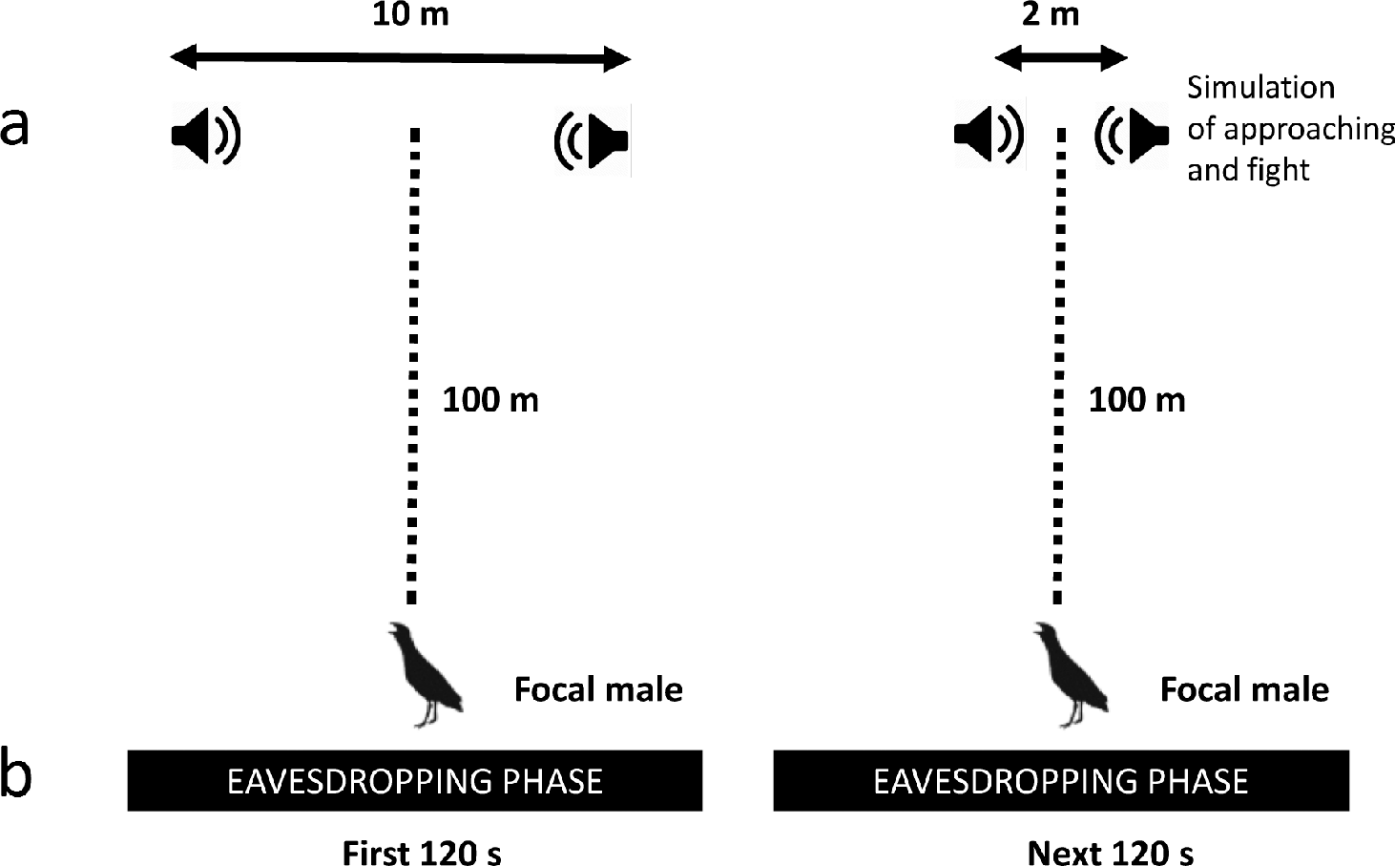
The experimental design of the eavesdropping phase: (a) speaker positions and (b) timeline.

**Fig 3:**
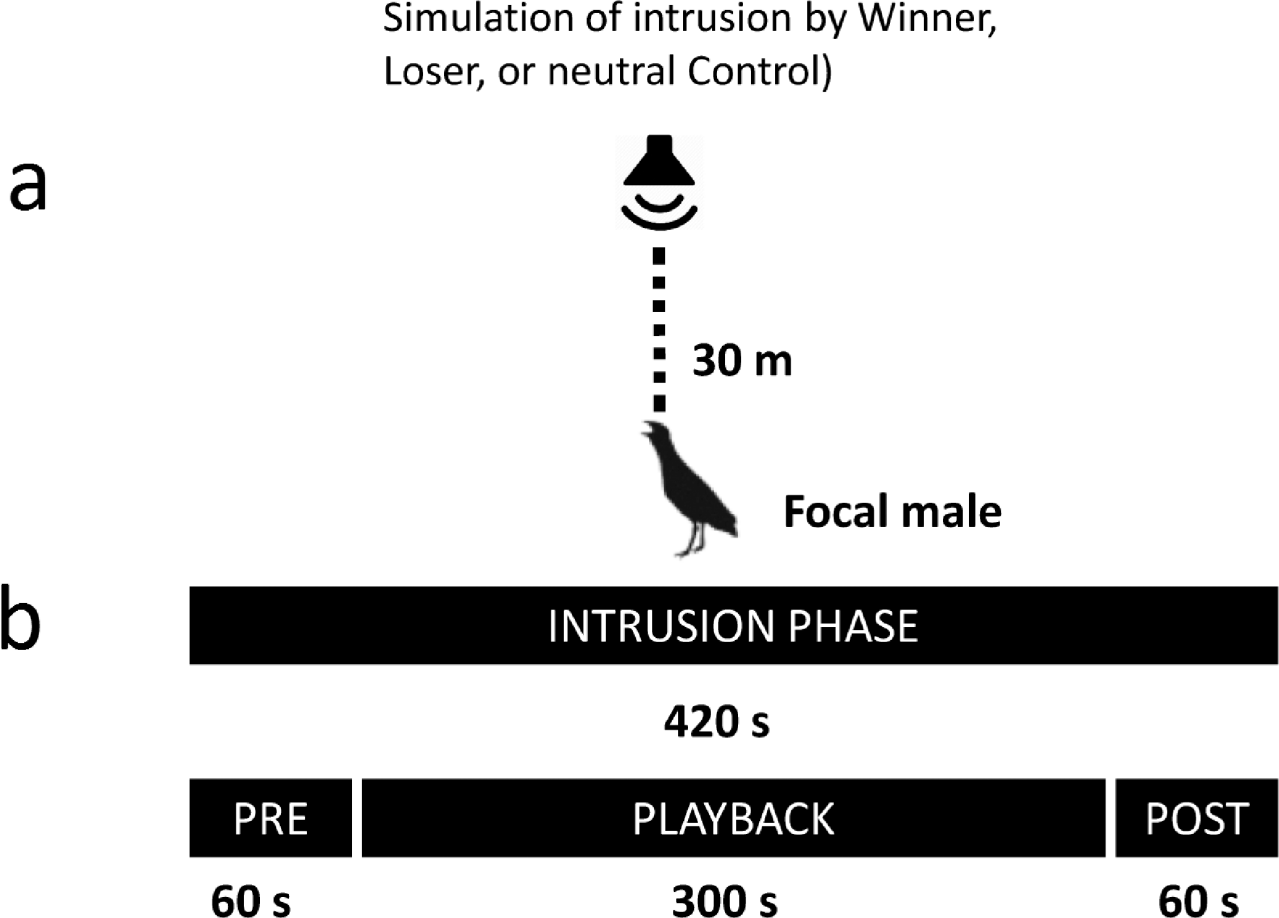
The experimental design of the intrusion phase: (a) speaker positions and (b) timeline.

Experiments were carried out between 2200 and 0230 h local time. Before each experiment, the loudspeakers were placed 0.20 m above the ground at a distance of 100 m from the calling subject. All experiments were conducted within an acoustic location system that consisted of an array of 4 omnidirectional microphones (Sennheiser K6/ME 62,Wennebostel, German), each of which was connected with a wireless signal transmitter (Sennheiser SKP 3000). Radio signals were received by a Sennheiser QUADPACK QP3041 sound receiver (with dedicated antennas and an antenna booster: Sennheiser EK 3241, A-2003 UHF, AB3-G) and finally recorded with an Edirol R-4 Pro 4-channel Portable Recorder and Wave Editor (Hamamatsu, Japan). Microphones were fixed approximately 1.2 m above the ground. The main idea of the experiment was to simulate natural aggressive or non-aggressive interactions between dyads of stranger males, on which the subject male could eavesdrop. Then, we simulated an invasion into the territory of the focal male with a playback of the calls of one of the males involved in the simulated interaction. We performed three treatments with different groups of males: Winner (W), Loser (L), and Control (C), and each treatment consisted of (1) eavesdropping and (2) intrusion phases, which were always carried out in the same order. During eavesdropping (240 s), focal males were able to listen to interactions between male dyads that were simulated via a set of four loudspeakers located 100 m away. The eavesdropping phases of the Winner and Loser treatments were exactly the same length. At the beginning, broadcast calls of simulated males were played back for 120 s from two loudspeakers separated by 10 m. Then, we simulated males approaching each other by silencing the first pair of loudspeakers and beginning playback from a second pair placed only 2 m apart for the next 120 s. At the end of the simulation of the W and L treatments, we played back soft calls, sounds of fight, sounds of flying away, and the final 60 seconds of broadcast calls from the Winner of the simulated conflict (Fig 2 and Fig 3). For the Control treatment, we only simulated a male-male interaction with broadcast calls at a distance of 10 m without the approaching or fight sounds or the final calls of the Winner. The set of experimental design was used on our previous experience with this species [32, 33, 34, 35, 36]. The intrusion phases of the experiments were started ca. 500 s after the eavesdropping event. During intrusions, we simulated the entrance of a male into the territory of the focal bird (starting 30 m from the focal male) using the playback of calls from one of the males in the eavesdropping phase (Winner, Loser or neutral, Fig 2). The intrusion phase lasted 420 s and consisted of the following stages: (1) 60 s of silence (PREPLAY) followed by (2) eight 20-s broadcast call playbacks separated by seven 20-s silence intervals (PLAY) and, finally, (3) 60 s of silence (POST) (Fig 3). We only initiated the second stage if the focal male was calling and was recorded during the PREPLAY phase. Calls during the PLAY stage consisted of an alternating playback with 20-s gaps for two reasons. Firstly, the playback used in this study reflected natural male-male interactions, and secondly, by avoiding strong overlap of the male calls and playback, we were able to more easily measure some response variables. Males were randomly assigned to the experiments and treatments, and the calls samples used in the experiments were 48 kHz / 16 bit PCM wave files played back with Creative Zen players (Creative Labs Ltd., Dublin, Ireland) and wireless MIPRO MA-101 (Chiayi, ROC Taiwan) loudspeakers with 30-W amplifiers and a frequency range of 60–15,000 Hz.

### (c) Preparation of call stimuli

The calls used in the experiments were recorded from the local population. In selecting calls for playback preparation, the priorities were high-quality recordings and the use of non-neighbour calls. The rhythm of a call is one of the most important factors affecting the response of rivals [33], since it signals aggression. When necessary, we changed the natural rhythm of the calls to fulfill the requirements of the experiment. Call rhythm was defined by Osiejuk et al. [32] as the ratio of the duration of Interval2 to the sum of the durations of Syllable1, Interval1 and Syllable2 (Fig 1). Additionally, call rhythm was calculated for each recording, and in our study call rhythm ranged from 0.53 to 1.10. We changed the rhythm by increasing or decreasing the duration of the silence in Interval2 to match a rhythm equalling 0.85 [33, 34]. All broadcast call playbacks were digitally prepared to match an amplitude of 96± 5 dB SPL at 1 m (natural level with a mean of 96 dB and a range of 80–101 dB), and soft calls were prepared to match 71 ± 5 dB SPL at 1 m. All of the sound processing was performed with Avisoft SASLab Pro 5.2.x (Avisoft Bioacoustics, Berlin, Germany). In each trial, we used broadcast calls obtained from two different males, and each was used only once. Syllable structure was not manipulated in any way, and we assumed that all information about identity remained unchanged. We also used soft calls and flight sounds (flapping wings or contact with vegetation) from our earlier recordings. As soft calls are relatively rare and difficult to record, we used a randomized sequence of the best quality recordings from different individuals for the playback.

### (d) Response measures

The strong response of male corncrakes to playback is well known [33]. Typically, calling males stop vocalizing with broadcast calls, for at least some time, after the start of playback; then, they move on the ground or sometimes fly but not necessarily right in the direction of the speaker. Finally, responding males approach the speaker and produce soft calls just before attacking it. Therefore, we extracted the following measures of male responses to playback from an array of recordings and notes dictated by one of the observers into a separate recorder: (1) broadcast calling, (2) movement, (3) flight, (4) approaching the speaker, (5) soft calling, and (6) attacking the speaker. All the measurements were calculated on a binary scale, but in some analyses, we also used the numbers of soft calls and attacks on the speaker as a better reflection of response aggressiveness. Movements on the ground and flights were associated with noticeable changes in the position of a male, while approaching reflected a change in position relative to the loudspeaker(s). These measurements were presented from least to most aggressive. Based on the repeatability of this behaviour, we also calculated an overall response strength index (later RSI), which rated each male on a scale of 0 to 6 and was calculated by adding all the reactions that we observed during the experiment. On this scale, 0 means that males did not cease regular broadcast calling from their initial position and no other behaviour was observed. In contrast, a score of 6 means that males ceased calling, more or less directly approached the speaker and then finished with soft calls and an attack on the speaker see McGregor 1992 [40] for a rationale for using such a compound variable). The corncrake is an exceptionally good species for studying aggression because males do vigorously attack the speaker, allowing for easy and direct measurement, and not just a proxy, of aggressive motivation [34]. Therefore, we also used the number of attacks on the speaker during intrusion as the most important variable describing male aggressiveness.

### (e) Statistical analysis

**Fig 4:**
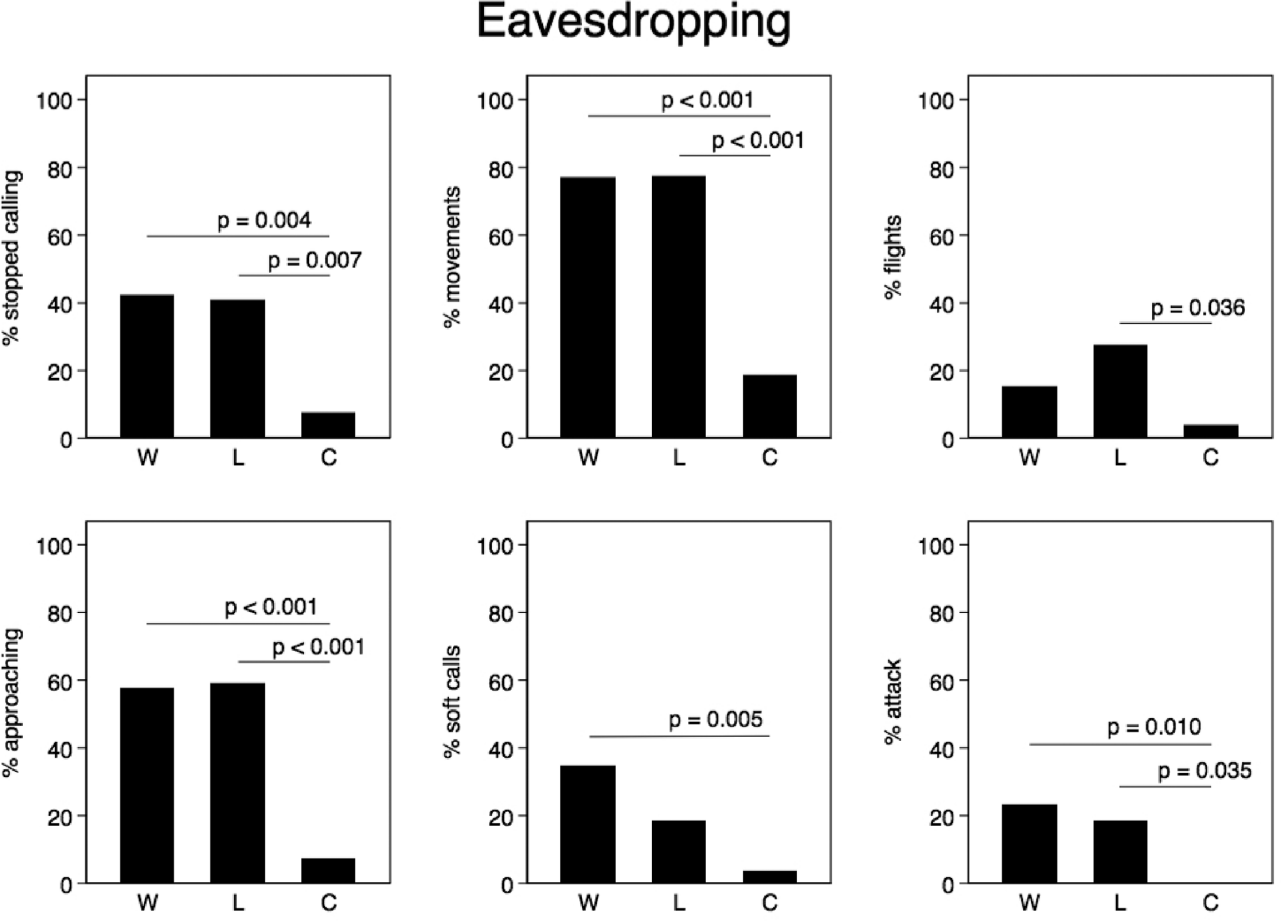
Response of male corncrakes during the eavesdropping phase.

**Fig 5:**
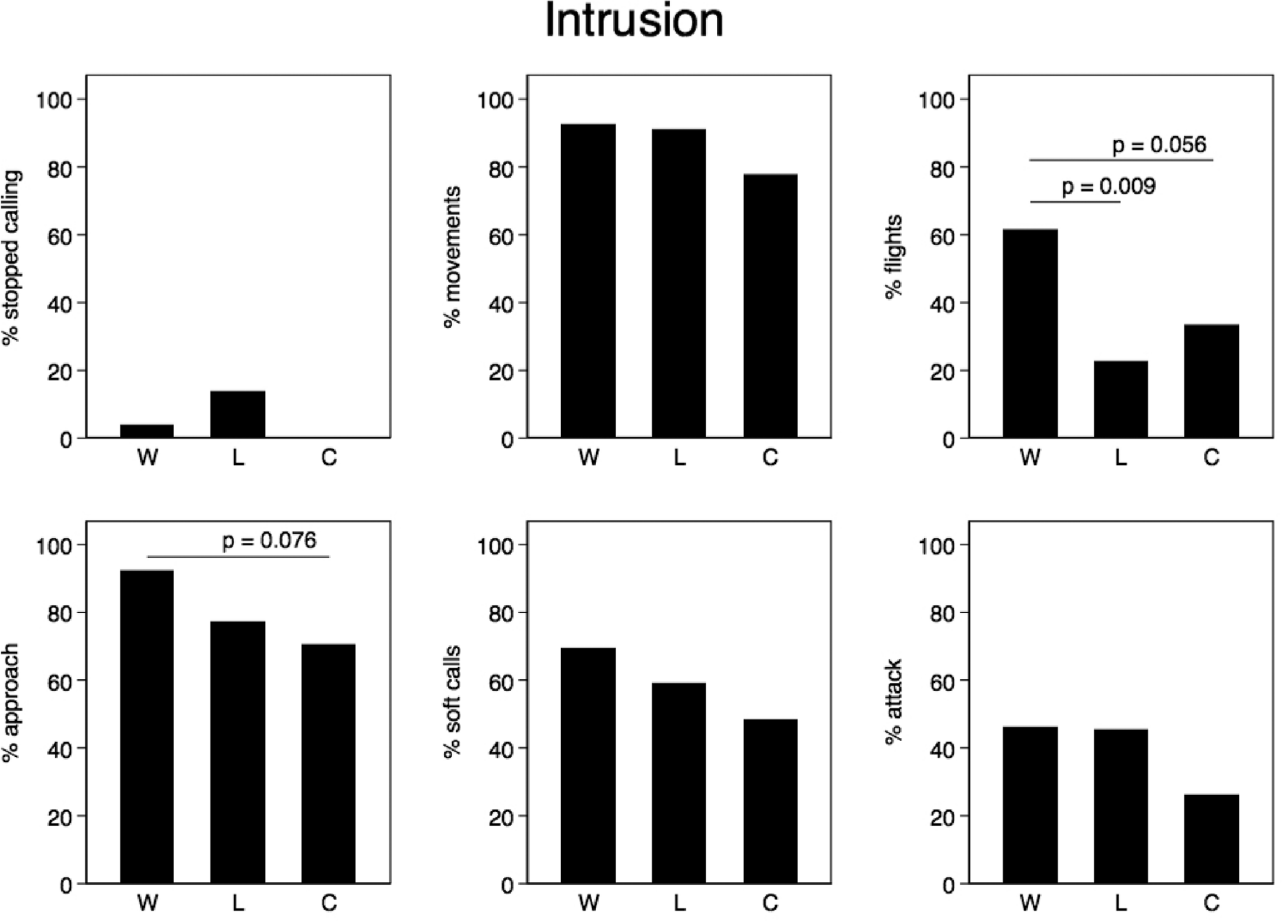
Response of male corncrakes during the intrusion phase.

To analyse the differences in the response between the experiments we used Fisher’s exact tests. The simple response measures (broadcast calling, movement, flight, approaching the speaker, soft calling, attacking the speaker) were treated as binary variables. This approach, together with a histogram of the frequency of particular response categories, provided a good overview of the behaviour of the birds during the experiments (Fig 4 and Fig 5). In more advanced analyses, we used the RSI and the number of attacks on the speaker as dependent variables and various generalized linear models (later GLM) to allow for the inclusion of more predictors and covariates (treatment, day in the season, hour after sunset, and male behaviour before intrusion began [e.g., rhythm of a male call before the experiment, cessation of calling during eavesdropping, etc.]). To model the predictors affecting the RSI, we used a GLM with a Poisson distribution, which best fitted the overall response of the males. To model the effect of treatment on the number of attacks, we used a GLM with a negative binomial distribution, which is especially useful for discrete data when the sample variance exceeds the sample mean [41] as was the case here (Eavesdropping phase: mean number of attacks = 0.7, variance = 10.0; Intrusion phase: mean number of attacks = 3.2, variance = 54.5). An information theoretic approach was used to compare candidate models on the basis of Akaike’s Information Criterion (AICc) corrected for small sample sizes, and models were ranked by ΔAICC, which is the difference between the best (lowest AICc value) and the AICc value of every other model. In the results section, we only present the best fitted models with ΔAICC < 2 [42]. Means are presented with ± SE unless stated otherwise. All the statistical analyses were two–tailed and were performed using STATA v. 14.2 (StataCorp, College Station, TX, USA) and IBM SPSS Statistics v. 24 (IBM Corp, Chicago, IL, USA).

## Results

### (a) Sample size

We tested a total of 75 males. Twenty-six received the Winner treatment; 22 received the Loser treatment; and 27 received the Control.

### (b) Behavioural response

#### (i) Eavesdropping phase

We found that focal males often started responding to simulated interactions during the eavesdropping phase of the experiment, especially in the W and L treatments. A high proportion of the males stopped calling (21%), approached the speakers (40%), and some even finished with soft calls (14%) and physical attacks on the speakers (13%) before the start of the intrusion phase (Fig 4). We found a clear pattern of difference in response between the two treatments (which were identical during the eavesdropping phase) and the Control. Males who listened to aggressive interactions (treatment W and L) responded more strongly than those who listened to neutral counter-calling that did not end with a fight (Control). We found that more males ceased calling during eavesdropping in both treatments than in the Control, and a similar pattern was found for movement, approaching, soft calls and even attacks (see Fig 4 for an illustration and the results of Fisher’s exact tests). Consequently, the overall measure of aggressive motivation, the RSI, during the eavesdropping phase was significantly affected by treatment (for models see Table 1) (GLM, treatment effect, estimate ± SE: -0.65 ± 0.12, *z* = -5.61, *p*< 0.001); it was higher during treatment W (2.5±0.31, Poisson exact 95% CI: 1.93–3.19) and treatment L (2.4 ± 0.33, Poisson exact 95% CI: 1.80–3.15) than during the Control (0.41 ± 0.12, Poisson exact 95% CI: 0.20–0.73). We found no significant effect of the call rhythm before the experiment, day or hour on the RSI (all *p*> 0.12). During the eavesdropping phase, 10 males attacked the speaker under the treatments (W and L combined), but none attacked under the Control. We found no significant difference in the number of attacks between those groups (W+L and Control) of males (GLM, *z* = -0.34, *p* = 0.736).

**Table 1:** Models with highest the probability (Δ AIC_C_ < 2) assessing variation in the corncrake males’ response to playback during eavesdropping and intrusion phase of experiments. The Akaike weight (*w*_*i*_) was calculated on the basis of Akaike’s Information Criterion corrected for small sample size. Predictors included: Treatment; Day of season; Hour after sunrise; Rhythm of call before the playback start. Estimates with standard errors and significance for predictor variables used in GLM models with highest probability (Δ AIC_C_ < 2) assessing variation in the corncrake males’ response to playback.

Model	AIC_C_	Δ AIC_C_	*w*_*i*_	Estimate ± SE and *p* values for predictor variables		
				Treatment	Day	Rhythm
**RSI** (Eavesdropping phase)						
Treatment + Day				-0.67 ± 0.117	0.02 ± 0.011	
	263.74	0.00	0.39	z = -5.70	z = 1.50	
				*p*< 0.001	*p* = 0.132	
						
Treatment				-0.65 ± 0.117		
	263.83	0.09	0.37	z = -5.61		
				*p*< 0.001		
						
**Number of attacks** (Eavesdropping phase)						
Treatment				-1.71 ± 0.321		
	157.29	0.00	0.58	z = -5.32		
				*p*< 0.001		
						
**RSI** (Intrusion phase)						
Treatment				-0.18 ±0.079		
	279.83	0.00	0.51	z = -2.27		
				*p* = 0.023		
						
**Number of attacks** (Intrusion phase)						
Treatment + Rhythm				-0.33 ± 0.161		3.77 ± 1.098
	360.24	0.00	0.83	z = -2.03		z = 3.34
				*p* = 0.042		*p* = 0.001

#### (ii) Intrusion phase

During the intrusion phase of the experiments, we observed a stronger male response than in the eavesdropping phase (Fig 5). Almost all the birds approached the speaker (80%). Around half of them used soft calls (40%), and a larger proportion (compared to the eavesdropping phase) finished with an attack on the speaker (39%). We found no significant differences (with one exception, see Fig 5) in response between treatment W, treatment L and the Control when treating each behavioural response category as a binary response. However, we found that treatment significantly affected the overall measure of aggressive motivation, the RSI (GLM, treatment effect, estimate ± SE: -0.18 ± 0.08, *z* = -2.27, *p*< 0.023) and was slightly higher in treatment W (3.6 ± 0.37, Poisson exact 95% CI: 2.95–4.47) and treatment L (3.1 ±0.37, Poisson exact 95% CI: 2.40–3.92) than in the Control (2.5 ±0.31, Poisson exact 95% CI: 1.99–3.23). All the other analysed models exhibited a poorer fit and did not indicate significance effects of call rhythm before the experiment, day or hour (all *p*> 0.34). We also found that focal males conducted significantly more attacks (for models see Table 1, GLM with a negative binomial distribution, treatment effect, estimate ± SE: -0.32 ± 0.16, *z* = -1.97, *p* = 0.049) on the speaker when we invaded the territory with Winner (4.6 ± 1.78, 95% CI: 1.1–8.2) or Loser calls (4.7 ± 2.17, 95% CI: 0.4–9.1) compared to the Control (2.4 ± 1.07, 95% CI: 0.3–4.5). The best model included the predictors of treatment effect (estimate ± SE: -0.32 ± 0.16, *z* = -2.03, *p* = 0.042) and call rhythm before the playback (rhythm effect estimate ± SE: -3.77 ± 1.10, *z* = 3.43, *p* = 0.001). The relationship between the call rhythm before the experiment and the number of attacks during the intrusion phase had quadratic characteristics. The strongest response, as indicated by the number of attacks, was given by males who signaled with a rhythm close to the one used for playbacks (see Materials and methods section).

### (c) Comparison of the response after eavesdropping with the response to neighbours and strangers (NSD) in a classical experiment

In the case of soft call production, we found that responding males in the intrusion phase of all experiments used soft calls significantly more frequently than in the neighbour treatment in the study by Budka and Osiejuk [36] (S1 Table). The results of the comparison of intrusion phases after eavesdropping with the stranger treatment from the NSD experiments were nearly identical, but we found no significant difference between the Control and stranger treatments in this study (S1 Table). In the case of attack occurrence, we found no significant differences between the neighbour treatment and Control (S2 Table). Under the stranger treatment, we found no significant differences among any of our experiments after the eavesdropping phase (S2 Table). These comparisons indicate that males responding to playback after earlier eavesdropping had a stronger response to intrusions by strangers than to neighbours without earlier eavesdropping.

## Discussion

Determining how animals settle conflicts is fundamental to understanding the evolution of resource defence behaviour [1], and previous studies of territorial defence strategies in birds are classical examples that have enriched our knowledge of the mechanisms and functions of acoustic signals in territoriality. Fundamental findings with extensive experimental support indicate that acoustic signals are used to keep rivals at a distance [27], and they also support the idea that acoustic signals act as identity cues, encoding crucial information for addressing a particular receiver and for appropriate signal exchange [5]. Thus, assessing the distance to an intruder and individual recognition constitute a baseline for making territory defence efficient and economically justifiable [43, 44, 45]. An important, and likely the most reliable, source of information about opponents in territorial systems is eavesdropping on actual signal exchange and conflicts between potential rivals [4]. Social eavesdropping provides additional information about the abilities or motivation of opponents and affects territory defence decisions of certain bird species following simulated interactions [19, 20, 26, 21, 22, 24, 46]. Although there have been many studies, our understanding of the processes involved in social eavesdropping and its commonness among bird taxa remains limited. A relatively small number of species have been thoroughly tested, and there is a bias toward studies of songbirds (*Oscines*). As there is tremendous vocal diversity among birds as well as different learning patterns and cognitive abilities, we may expect that social eavesdropping evolved under various selective pressures. The purpose of this study was to broaden the knowledge about social eavesdropping through experimentation with a model, non-learning, bird species, for which acoustic communication in territorial defence is crucial because the usefulness of other communication channels is poor due to its nocturnal lifestyle. In addition, corncrakes behaviour in aggressive interaction is very straightforward and relatively easy to describe. Males either attack or escape from aggressors. Our results revealed the existence of eavesdropping in male corncrakes. We found that these birds pay close attention to the vocal interactions occurring within the proximity of their territories. Males were more likely to initiate a response and showed a stronger response when eavesdropping on aggressive interactions compared to eavesdropping on neutral counter-calling. An aggressive interaction appeared to be such a strong stimulus that some males approached the speaker and attacked it as early as the eavesdropping phase. Males usually respond to playbacks of a call (of a single intruder) if the distance to the speaker is much closer (±20–30 m) than in our experiments (100 m) [33, 34, 47, 38]. Difference in the response to eavesdropped individuals of different status (Winner, Loser) is one of the main predictions of the social eavesdropping hypothesis [4] but we found no significant differences in the response to the Winner or Loser of a previous interaction.

### Learning abilities

Most of the bird species studied with respect to social eavesdropping have been songbirds whose learning abilities are well known [28], especially in terms of the acquisition of new vocalizations [28]. Therefore, the lack of a different response to the calls of Winners and Losers could only be attributed to weaker cognitive abilities in the corncrake if simpler and inherited calls are also supported by less sophisticated sensory or signal-processing systems, but there are no indications that this is the case. Other in-depth studies of non-passerines have revealed strong cognitive abilities for discrimination and associative learning despite signs of a simpler brain structure [48, 49], and strong cognitive abilities have also been demonstrated in rails [50]. Moreover, similar to humans, the cognitive systems underlying vocal production and auditory recognition memory in birds are known to be supported by distinct brain regions [51]. To our knowledge, there is only one previous study that focuses on eavesdropping in another non-learning bird species, the little blue penguin [27] which is a colonial bird that nests in burrows and uses both acoustic and visual signalling. Mouterde et al. [27] found support that post-conflict ‘triumph displays’ act as an advertisement of victory for social eavesdroppers, and they experimentally showed that males exposed to Winner and Loser calls respond differently by means of heart rate and threatening behaviour. This study suggests that the ability to learn vocal signals is not a limiting factor in eavesdropping, and additional support came from a study assessing the recognition of the meaning of a new signal by the corncrake [35]. It was demonstrated that corncrake males could comprehend a new association between calling rhythm and the approach of an intruder [35], so despite the simple vocal structure, corncrakes seem to be able to show dynamic associations between a signalling pattern and its meaning, which is essential in a social eavesdropping context.

### Social eavesdropping or just eavesdropping

The results of our study are comparable (in terms of signal propagation conditions) to earlier research on the common nightingale (*Luscinia megarhynchos*). Males of this species sing extensively at night, so their decisions concerning territorial defence likely rely solely on acoustical perception [52]. The authors of that study demonstrated that territorial nightingales eavesdropped on the vocal interactions between neighbours and unfamiliar intruders and used the responses of neighbours as a cue for tuning their own response. In particular, nightingales responded strongly when their neighbours exhibited a more intense singing performance. Naguib et al. [52] also concluded that the eavesdropping behaviour of neighbours acted as an early warning system for threats to territorial integrity. In contrast to the nightingale study, we did not use the calls of neighbours in our experimental design. We always simulated an interaction between two unfamiliar males, so the differences in the response could only be attributed to the nature of the simulation (Winner vs. Loser or neutral counter-calling) and the identity cues in the calls of males that were used later in the intrusion phase. We found no significant difference in the response to the intrusion of a Winner or Loser and a weaker response to the Control playback of a neutral-male call. Therefore, it seems that corncrakes more frequently match the strength of their response to the context of the eavesdropped interaction than to the status and identity of the intruder. Support for this explanation came from a study of the red-capped cardinal, *Paroaria gularis*[53], in which territorial cardinals were able to localize intruders if the intruder had previously been chased away from the territory of a neighbour. To some extent, our experiment simulated a similar situation and suggests that corncrakes pay close attention to interactions within the neighbourhood and consider, as did the red-capped cardinals, adjacent territorial conflicts to be dangerous. Thus, the stronger response to treatments than to the Control (including the eavesdropping phase) in our study seems to be an attempt to join and resolve a conflict within a limited area. The study by Fitzsimmons et al. [46] on black-capped chickadees also demonstrated that territorial males responded more intensively to aggressive than to submissive playback outside their territory, and they also found that birds closer to the simulated interaction increased their song output more than those separated from the speaker by more than one territory. There was no effect of male dominance rank on playback response. The design of the black-capped chickadee experiment was very similar to that of our experiment, and some of the results are consistent with our findings including eavesdropping on the interactions between unknown individuals outside the territory.

Despite clear eavesdropping on interactions, male corncrakes generally did not differentiate their response to Winner and Loser playbacks during intrusions. We suggest two potential explanations for this observation. First, a potential functional explanation could be related to strategic decision making by focal males; i.e., respond strongly to any unknown intruder that was recently involved in an aggressive interaction. Our experiments were conducted in the context of conflict among males requiring the same types of resources to increase their fitness, and in such situations, the outcome of a contest depends on the fighting ability (or resource-holding) ability, potential, motivation and experience of males. Earlier studies of corncrakes revealed that they signal their fighting ability through the rhythm of their broadcast call, which is an arbitrary character whose honesty is maintained by the retaliation rule [33, 34]. It is difficult to directly assess whether call rhythm only reflects the probability of successfully defending a resource or whether the motivation to protect the resource, which reflects its value, also affects the rhythm. It seems probable that the baseline rhythm directly results from fighting ability but is also modified by the current motivation. Rhythm has been found to decrease slightly during the night and is much more variable within a season [32], but information is lacking as to whether a male has experience from prior interactions and from eavesdropping on contests between third-party individuals. We had no knowledge of the experience of focal males from natural interactions with neighbours or floaters, so we were only able to focus on the partial experience from simulated interactions. Animals are known to be better at decision making if they can assess their chance of winning against a particular opponent. In our case, the only information came from eavesdropping on interactions and was likely based on a rule that if an animal has won a previous fight, it is more likely to win a subsequent contest (and the converse for a loser) [54, 55]. Therefore, it seems that male corncrakes did not use information about the identities of Winners and Losers and did not tune their responses to the results of the intruder’s previous fight. Therefore, the question is whether they failed to use eavesdropped information or if they were unable to extract such information? The last possibility leads to the second explanation for the lack of different responses to previous Winners and Losers as being mechanistic due to the process of individual discrimination. Male corncrakes have been experimentally shown to discriminate between neighbours and strangers [36], but the mechanism underlying this discrimination is not yet fully understood. The most obvious individually specific corncrake call characteristics, namely PPD, were recently shown to be unimportant for neighbour-stranger discrimination by male birds [45] despite being useful for human observers discriminating between individuals using bioacoustics software [37, 33, 34, 38]. The formant variation in calls seems to be the next candidate for carrying identity cue information [56], but experimental support is still required. Regardless of the mechanisms, corncrakes that must discriminate between neighbours and strangers under natural conditions may be exposed to thousands of neighbour call renditions within a few hours [57, 37], which may considerably facilitate the differentiation between a neighbour and a stranger. In our experiments, males eavesdropped on interactions between strangers for only a few minutes, which may have hindered the later differentiation in their response to Winners and Losers. However, as mentioned earlier, experiments on learning have demonstrated the quick learning abilities of corncrakes [58].

### Eavesdropping as a preparation for fight

We compared the use of soft song and speaker attacks in this study with the results of earlier experiments testing (with positive results) the neighbour-stranger discrimination ability of corncrakes [36]. For this comparison, we used the responses from the intrusion phases from this study, which had the same design as those from Budka and Osiejuk [36] but were preceded by an earlier eavesdropping phase; therefore, the basic difference was that the birds being compared either did or did not listen to an earlier interaction. It was clear that eavesdroppers were using soft calls more frequently than males responding to neighbours or strangers (S1 Table). Furthermore, eavesdroppers attacked speakers more frequently in response to call by stranger males than to neighbouring males, and there was no difference in attack frequency between eavesdroppers and males responding to strangers (S2 Table). This comparison demonstrates that eavesdroppers are more likely to prepare for a fight (soft song use) than non-eavesdroppers and that stranger intrusion is always treated as a highly dangerous situation by the study species.

## Conclusions

We found that males of a non-learning, nocturnal rail species eavesdrop on signal exchange between rivals outside their territories. Focal males often started responding to distant rival interactions during the eavesdropping phase, which was not observed if playback of a single male was presented outside of a territory. This suggests that eavesdropping behaviour acts as an early warning system for threats to territorial integrity. We also found that, as a rule, males responded strongly in the intrusion phase of the experiments, but the response to playback of males from the Control (i.e., after neutral counter-calling) was significantly weaker than that to both treatment groups (aggressive interaction). We found no support for a difference in the response to Winners and Losers of simulated interactions, and we also found that the strongest response, as represented by the number of attacks, was by males who called with a rhythm that was comparable to the rhythm of the playback (i.e., indicating a similar motivation). To sum up, our study revealed that male corncrakes eavesdrop on the signal exchange between conspecifics outside their territories and that the strongest factor affecting their response was the character of the eavesdropped interaction (aggressive or neutral). We did not find strong support for different responses to Winners and Losers, which is likely due to a territory ownership strategy of responding strongly to unknown males that took part in an earlier aggressive encounter.

## Supporting information

S1 Table Comparison of soft calls use in experiments from this study and neighbour-stranger discrimination study (Budka and Osiejuk 2014). Proportions are indicated as number of experiments with soft calls/number of all experiments, and this study vs NSD study. Significantly larger proportions in **bold.**
(PDF)

S2 Table Comparison of attacks occurrence in experiments from this study and neighbour-stranger discrimination study (Budka and Osiejuk 2014). Proportions are indicated as number of experiments with attacks/number of all experiments, and this study vs NSD study. Significantly larger proportions in **bold.**
(PDF)
