# Supplementary material for: A nocturnal rail with a simple territorial call eavesdrops on interactions between rivals
Source: PLoS One. 2018 May 18;13(5):e0197368. doi: 10.1371/journal.pone.0197368 (PMC5959188; doi:10.1371/journal.pone.0197368)
Supplement: S1 Table — Proportions are indicated as number of experiments with soft calls/number of all experiments, and this study vs NSD study. Significantly larger proportions in bold. (PDF) [file pone.0197368.s001.pdf]

Experiment 1 (100m)	Neighbour intrusion Proportions	Neighbour intrusion Fisher's Exact test	Stranger intrusion Proportions	Stranger intrusion Fisher's Exact test
Winner intrusion	18/26 vs 10/43	**p < 0.001**	18/26 vs 13/43	**p = 0.003**
Loser intrusion	13/22 vs 10/43	**p = 0.006**	13/22 vs 13/43	**p = 0.034**
Control	13/27 vs 10/43	**p = 0.039**	13/27 vs 13/43	p = 0.203
