# Supplementary material for: A nocturnal rail with a simple territorial call eavesdrops on interactions between rivals
Source: PLoS One. 2018 May 18;13(5):e0197368. doi: 10.1371/journal.pone.0197368 (PMC5959188; doi:10.1371/journal.pone.0197368)
Supplement: S2 Table — Proportions are indicated as number of experiments with attacks/number of all experiments, and this study vs NSD study. Significantly larger proportions in bold. (PDF) [file pone.0197368.s002.pdf]

Experiment 1 (100m)	Neighbour intrusion Proportions	Fisher's Exact test	Stranger intrusion Proportions	Fisher's Exact test
Winner intrusion	12/26 vs 4/43	***P*< 0.001**	12/26 vs 13/43	*p* = 0.206
Loser intrusion	10/22 vs 4/43	***p* = 0.003**	10/22 vs 13/43	*p* = 0.277
Control	7/27 vs 4/43	*p* = 0.092	7/27 vs 13/43	*p* = 0.790
